# Radiotherapy alone or with chemotherapy in the treatment of small-cell carcinoma of the lung: the results at 36 months. 2nd report to the Medical Research Council on the 2nd small-cell study.

**DOI:** 10.1038/bjc.1981.245

**Published:** 1981-11

**Authors:** 

## Abstract

This report compares the results at 36 months for 121 patients treated with radiotherapy alone (R) and 115 with radiotherapy followed by 3-drug chemotherapy (RC) for small-cell carcinoma of the lung of "limited" extent. The RC patients had an increased survival (P = 0.009 by log-rank test). The median survival was 25 weeks for the R patients and 43 weeks for the RC patients, but at 36 months, only 4 (3%) of the R patients and 5 (4%) of the RC patients were still alive. There was evidence of recurrence of the primary cancer in 41 (35%) of the 117 R and 35 (32%) of the 110 RC patients who died. Distant metastases were more frequent in the R series, being reported in 99 (82%) compared with 82 (71%) of the RC patients (P less than 0.05 by log-rank test). The numbers of R patients alive and considered free of metastases were 10 (8%) at 12 months, 3 (2%) at 24 months and 3 (2%) at 36 months; the corresponding figures for the RC patients being 30 (26%), 9 (8%), and 4 (3%).


*Br. J. Cancer* (1981) **44**, 611

### RADIOTHERAPY ALONE OR WITH CHEMOTHERAPY IN THE TREATMENT OF SMALL-CELL CARCINOMA OF THE LUNG: THE RESULTS AT 36 MONTHS

### 2nd REPORT TO THE MEDICAL RESEARCH COUNCIL ON THE 2nd SMALL-CELL STUDY

MEDICAL RESEARCH COUNCIL LUNG CANCER WORKING PARTY*

* Members: Prof. N. M. Bleehen (Chairman), Mr W. P. Cleland (until June 1979), Dr T. J. Deeley, Mr P. M. Fayers (from September 1977), Prof. W. Fox, Dr D. J. Girling (from October 1978), Dr L. E. Hill (Secretary until October 1978), Dr K. F. W. Hinson, Dr A. R. Laing, Dr J. R. Lauckner, Dr I. McHattie, Miss R. Tall (until September 1977).

Received 24 June 1981 Accepted 3 July 1981

**Summary.—This report compares the results at 36 months for 121 patients treated with radiotherapy alone (R) and 115 with radiotherapy followed by 3-drug chemotherapy (RC) for small-cell carcinoma of the lung of "limited" extent.**

**The RC patients had an increased survival ($P=0{\cdot}009$ by log-rank test). The median survival was 25 weeks for the R patients and 43 weeks for the RC patients, but at 36 months, only 4 (3%) of the R patients and 5 (4%) of the RC patients were still alive.**

**There was evidence of recurrence of the primary cancer in 41 (35%) of the 117 R and 35 (32%) of the 110 RC patients who died. Distant metastases were more frequent in the R series, being reported in 99 (82%) compared with 82 (71%) of the RC patients ($P<0{\cdot}05$ by log-rank test). The numbers of R patients alive and considered free of metastases were 10 (8%) at 12 months, 3 (2%) at 24 months and 3 (2%) at 36 months; the corresponding figures for the RC patients being 30 (26%), 9 (8%), and 4 (3%).**

SMALL-CELL CARCINOMA of the lung is usually treated by non-surgical methods. It is very sensitive to radiation and chemotherapy, and current management usually includes both (Hansen, 1980; Oldham & Greco, 1980). Although survival has been improved, the best drug combinations and the best ways of using radiation therapy still remain to be defined.

The present study, to which patients were admitted between March 1975 and April 1977, was designed to compare radiotherapy alone (R) with radiotherapy followed by chemotherapy with cyclophosphamide, methotrexate and CCNU (RC). The main findings at 12 months (MRC Lung Cancer Working Party, 1979) were that in the RC series, there was a significantly increased survival ($P=0{\cdot}002$ by log-rank test) distant metastases appeared later and less frequently, but adverse reactions were much more frequent and severe. In this report the results on all patients up to a minimum of 3 years are presented.

### PLAN AND CONDUCT OF THE STUDY

The plan and conduct of the study were described in detail in the first report (MRC Lung Cancer Working Party, 1979). The main points are summarized below.

### *Eligibility*

Patients aged 70 years or less were eligible if they had a previously untreated histologically or cytologically proven small-cell carcinoma (Kreyberg *et al.*, 1967) confined to the soft tissues of one hemithorax and the ipsilateral and contralateral scalene and lower cervical nodes, the cell type being confirmed by a single reference pathologist.

Requests for reprints to Miss J. S. Leadbeater, Medical Research Council, 20 Park Crescent, London W1N 4AL.

*Treatment*

Patients were randomly allocated to treatment with either radiotherapy alone (R), or radiotherapy followed by 3-drug chemotherapy (RC).

Radiotherapy consisted of a megavoltage midline dosage of 30 Gy given in 15 fractions over 3 weeks, or a suitable biological equivalent. There was an interval of 3 weeks between the end of the radiotherapy and the first pulse of chemotherapy.

Chemotherapy consisted of 10 alternating 3-drug and 2-drug pulses at 3-week intervals, though it could be stopped before the completion of 10 pulses or prolonged beyond 10 pulses if the patient's progress warranted it. Cyclophosphamide (500 mg/m$^2$) plus methotrexate (50 mg/m$^2$) were given by i.v. injection on each occasion, 10 mg of metoclopramide being included as an anti-emetic. CCNU (50 mg/m$^2$) was given orally on the first and alternating pulses thereafter, that is every 6 weeks for 5 pulses.

*Reports and investigations*

A report on each patient was completed pretreatment, at each attendance for treatment, monthly up to 18 months and then once every 3 months. These reports included information on the allocated therapy, additional palliative therapy, any adverse reactions encountered, and metastases.

RESULTS

A total of 253 patients were admitted from 16 centres in the U.K.; 17 were excluded (MRC Lung Cancer Working Party, 1979), leaving 236 (121 R, 115 RC) for analysis.

*Condition on admission*

The details of the patients' condition on admission were given in the first report (MRC Lung Cancer Working Party, 1979). The majority (72%) were male, 66% were aged 55–70 years, 58% were considered by their physician to be in "good" clinical condition, 51% were capable of normal or nearly normal activity (activity grades 1 or 2) and the respiratory assessment was

FIG.—Survival from allocation in 121 R and 115 RC patients.

normal or nearly normal (grades 1 or 2) in 50%.

*Survival*

The survival curves (Fig.) show no difference up to the end of the first 3 months (well after the end of the course of radiotherapy). Thereafter there was increased survival in the RC series ($P=0\cdot009$, log-rank test). At 12 months 22 (18%) of the 121 R patients were alive (95% confidence limits 12–26%), compared with 39 (34%) of the 115 RC patients (limits 25–43%) ($P=0\cdot009$). However, at 36 months the corresponding figures were only 4 (3%) and 5 (4%) respectively. The median survival was 25 weeks for the R patients (limits 22–34) and 43 weeks for the RC patients (limits 34–48).

Of the 4 R patients alive at 36 months, 1 died with metastases at 39 months, 1 is alive at 63 months, but with metastases, and 2 are alive, well and with no evidence of recurrence at 61 and 62 months, respectively. Of the 5 RC patients alive at 36 months, 2 died, 1 at 37 months with metastases, the other of cor pulmonale at 52 months with no evidence of cancer, 1 is alive at 53 months, but with metastases, and 2 are alive, well and with no evidence of recurrence at 38 and 50 months, respectively.

*Prognostic factors*

Age, weight, haemoglobin concentration, total white cell and platelet counts, clinical condition, grade of activity and respiratory assessment on admission, and sex were examined singly and in combination, using stratification of the log-rank test (Peto *et al.*, 1977) for their relationship to survival. The only factors found to be significantly related were age ($P=0\cdot03$) and clinical condition ($P=0\cdot03$). None of the factors affected the comparison between the R and RC series.

*Evidence of primary growth at death*

There was clinical or radiographic evidence of persistence, extension or recurrence of the primary cancer in 41 (35%) of the 117 R and 35 (32%) of the 110 RC patients who died during the 36 months. Such evidence was confirmed in 14 of the 18 R and 15 of the 24 RC patients who had a necropsy, but of these 29 patients, 22 (11 in each series) also had distant metastases.

*Metastases*

The Table shows the frequency of metastases during the 36 months; 82% of the 121 R and 71% of the 115 RC patients developed clinical evidence of distant metastases ($P<0\cdot05$ by log-rank test), a smaller difference than at 12 months, when the corresponding proportions were 79% and 57% ($P=0\cdot0005$). All the patients with metastases except 2 (both RC) had their metastases diagnosed during the first 24 months. The differences between the series for individual sites were small. At 12 months 10 (8%) of the R and 30 (26%) of the RC patients were alive and considered to be free of metastases, at 24 months 3 (2%) and 9 (8%), respectively, and at 36 months 3 (2%) and 4 (3%) respectively.

TABLE.—*Cumulative occurrence and site af distant metastases during* 36 *months*

| | R | | RC | |
|---|---|---|---|---|
| Site | No. | % | No. | % |
| Brain | 30 | 25 | 37 | 32 |
| Bone | 38 | 31 | 36 | 31 |
| Liver | 62 | 51 | 56 | 49 |
| Lymph nodes | 19 | 16 | 12 | 10 |
| Opposite lung | 15 | 12 | 16 | 14 |
| Other | 20 | 17 | 17 | 15 |
| Total patients with distant metastases | 99 | 82 | 82 | 71 |
| Total patients | 121 | 100 | 115 | 100 |

*Modifications to chemotherapy in the RC series*

Of the 101 RC patients who started their prescribed chemotherapy, 33 (33%) received it without modification, 19 of them completing the course and 14 dying during the course. A further 30 (30%)

had 1 or more doses omitted, delayed or modified because of adverse reactions, 19 of them eventually completing the course. The remaining 38 (38%) did not complete the planned course of chemotherapy, 22 because of adverse reactions, 15 because it was considered that the disease was not responding, and 1 because of default.

A total of 12 patients were still receiving the prescribed chemotherapy at 12 months because their disease was still considered to be responding. Chemotherapy was eventually stopped after the 12-month dose for 1 of these 12 patients, at 14 months for 3, at 16 months for 2, and at 17, 18, 20, 23, 26 and 27 months for the remaining 6 respectively. Of the 12, 4 died while still receiving chemotherapy. Of the remaining 8, 3 were alive at 36 months having received 16, 23 and 27 months of chemotherapy, respectively; 1 of the 3 died at 37 months, the other 2 are alive at 50 and 53 months respectively, the first with no evidence of recurrence, the second with metastases.

*Additional palliative treatment*

Of the 117 R and 109 RC patients who completed the prescribed radiotherapy, 60 (51%) of the R patients were given additional palliative therapy during the first 12 months, compared with 21 (19%) of the RC patients. For the 60 R patients, this was chemotherapy alone for 17 (15%), radiotherapy alone for 25 (21%), and radiotherapy and chemotherapy for the remaining 18 (15%). For all 21 of the RC patients it was radiotherapy alone.

During the second year a further 2 R patients had additional palliative treatment (one radiotherapy, the other radiotherapy and chemotherapy) as did 7 RC patients (1 radiotherapy, 6 radiotherapy and chemotherapy). During the third year, 2 other RC patients had palliative treatment (one radiotherapy, the other radiotherapy and chemotherapy). Thus, during the 36 months, a total of 62 (53%) of the R patients were given additional palliative treatment, compared with 30 (28%) of the RC patients ($P = 0{\cdot}0002$).

*Adverse reactions*

The adverse reactions reported during the first 12 months were described in detail in the first report (MRC Lung Cancer Working Party, 1979). In summary, 38 (32%) of the 118 R and 93 (83%) of the 112 RC patients who started treatment had adverse reactions, either to their allocated therapy or to palliative therapy, during the first 12 months ($P < 0{\cdot}0001$). The commonest reactions were nausea and vomiting, which occurred in 15 (13%) of the R and 79 (71%) of the RC patients ($P < 0{\cdot}0001$), and haematological reactions which occurred in 27 (23%) and 60 (54%) respectively ($P < 0{\cdot}0001$).

Only one patient in each series had any evidence of an adverse reaction for the first time after 12 months. The R patient had a total white cell count of 2200/mm$^3$ in the 25th month during palliative chemotherapy, and the RC patient had a platelet count of 98,000 in the 23rd month, while still receiving the allocated chemotherapy which had been continued without a break, but which was then stopped.

### DISCUSSION

There have been definite achievements in the management of small-cell carcinoma of the lung over the past few years. An optimistic assessment of the current situation suggests that up to 25% of patients with limited-stage disease may survive to 2 years as a result of intensive therapy (Oldham & Greco, 1980). However, the great majority of patients still die of their disease, often very rapidly.

The results of this study are clearly not as good as the best reported (reviewed by Hansen, 1980). There are probably several reasons for this, some of which are obvious and others are hypothetical. They include the extent of disease when treatment is started, and the choice of radiotherapy and chemotherapy regimens, and the sequence in which they are given. Age and clinical condition on admission influenced prognosis in the present study. Age has not consistently been found to influence prognosis in other studies; its influence was not great in the present study ($P = 0{\cdot}03$), so may have been a chance finding. Initial clinical condition probably correlates more closely with extent of disease, and other studies have reported an association between survival and clinical assessments.

The use of radiotherapy in the management of local disease has been general practice until recently (reviewed by Bleehen, 1979; Salazar & Creech, 1980; Hansen, 1980). The first MRC small-cell study (Fox & Scadding, 1973) demonstrated the superior results of radiotherapy alone over those of surgery alone, though this conclusion has subsequently been questioned (Levison, 1980) for a very selected series of patients whose disease is considered to be sufficiently localized for them to be treated with radiotherapy followed by surgery (usually pneumonectomy).

The radiation dosage to the local disease has varied very considerably in different series. Thus Salazar & Creech (1980) quote a mean total dosage of 45 Gy (range 42–55 Gy) in 446 patients treated by 10 different groups. The dosage selected for the present study was considerably lower, and was chosen in a deliberate attempt to keep radiation morbidity to a minimum, while permitting chemotherapy. The fact that about one third of all the patients treated in this study had clinical or radiographic evidence of persistence, extension or recurrence of the primary tumour, suggests that this radiation dosage was inadequate. However, there is little evidence in well conducted controlled studies, that higher radiation dosages do in fact improve local control or survival (reviewed by Bleehen, 1979, and by Hansen *et al.*, 1980). Also, in the present study, most of the patients with evidence of cancer at the primary site at death, had distant metastases; it is therefore doubtful whether a higher radiation dosage would have improved the results.

Radiotherapy has also been advocated as a method of controlling occult metastasis. This may involve whole-body or hemi-body irradiation (Dawes, 1980; Salazar *et al.*, 1980) or, more usually, elective radiation to common metastatic sites, in particular the brain (Johnson *et al.*, 1976; Livingston, 1979; Hansen, 1980). While there is evidence that the incidence of CNS metastases may be reduced by such procedures, there is no evidence in randomized studies of an associated prolongation of survival (Hansen, 1980).

The choice of the chemotherapy regimen has been discussed in the first report on this present study (MRC, 1979) and was based on the report by Hansen *et al.* (1976) of its efficacy. The results of the present study confirm its activity, in that its addition to the radiotherapy significantly improved survival. However, the inclusion of the drug CCNU was regarded unfavourably by many patients and their clinicians because of the associated side effects. The absence of any reduction in the incidence of CNS metastases has raised questions about its ability to control them.

Numerous other chemotherapy regimens have been reported, with varying results (Hansen, 1980; Oldham & Greco, 1980). Of relevance to the present report are the results of a randomized trial by Hansen *et al.* (1980) using a very similar drug regimen and a not dissimilar total radiation dosage. Their staging investigations were more rigorous, and are therefore likely to have allowed the inclusion of a smaller proportion of patients with unrecognized "extensive" disease than in the present study, in which isotopic scans, marrow examination and peritoneoscopy were not mandatory. The median duration of survival reported by Hansen *et al.* (1980) was, however, very similar to that reported here, namely 310 days compared with 300 days (43 weeks), and the proportion of patients alive at 18 months was the same (13% in both series). It is of interest that no improvement in survival was seen by the Danish group when they added irradiation of the brain, adrenals and upper retroperitoneal lymph nodes.

Better results, but in much smaller series of patients, have been reported, with a

2-year survival of ~25% (Oldham & Greco, 1980). If differences in patient selection are not the main or only reason for this difference, our failure to achieve such results may then relate to the choice of chemotherapy regimen. Evidence for this may be deduced from the study reported by Cohen *et al.* (1977) in which the 3-drug regimen (cyclophosphamide, methotrexate and CCNU) at similar dosages was compared with the same drugs given at approximately double dosages. Toxicity was considerably greater with the higher dosages, but so was the median survival. Other drugs with activity against small-cell lung cancer such as vincristine, Adriamycin, and the podophyllin derivatives, VP16–213 and VM26, are now being included in various combinations, and may produce better maintained survival.

In the present study, the sequence in which the two treatment modalities were combined was selected with the aim of controlling local disease with radiotherapy first before giving systemic treatment to occult disease. This sequence might be criticised as permitting the early development of metastases during the 6 weeks before chemotherapy. Data in favour of this concept have been reviewed (Bleehen, 1980; Salazar & Creech, 1980) but are inconclusive. This question of drug-radiation sequence is being formally tested in the current (3rd) MRC small-cell study, and in a Swiss study (Alberto, personal communication).

Finally, the necessity for radiotherapy in the primary management of the disease is now being questioned (reviewed by Hansen, 1980). In 2 large randomized series of patients (total 259) median survival was not improved by the addition of radiotherapy to the selected drug regimens (Hansen *et al.*, 1979; Fox *et al.*, 1980). It may well be that, with further improvements in chemotherapy, radiotherapy will have no place in the primary treatment. Alternatively, improved radiation therapy with radiosensitizers or particle therapy may, in the future, produce better control of local disease and enhance the systemic results of improved chemotherapy. Both these possibilities remain a matter for speculation. At present, the results of treating small-cell carcinoma of the lung remain poor, and considerably more effort with carefully documented randomized studies, after innovative pilot studies, is required.

The following physicians, radiotherapists and pathologists co-operated in the study:

Bristol: Dr H. Eckert, Mr N. C. D. Pizey; Cambridge: Dr V. Barker, Professor N. M. Bleehen, Dr P. G. I. Stovin, Dr C. R. Wiltshire; Cardiff: Dr G. Anderson, Dr S. G. Cotton, Dr B. Davies, Dr T. J. Deeley, Dr G. S. Kilpatrick, Dr R. Seal, Dr A. Seaton, Dr P. Smith; Durham: Dr J. E. Ennis, Dr G. S. Graham, Dr A. L. Hovenden, Dr J. S. Law; Glasgow: Dr J. C. J. L. Bath, Dr R. A. Burnett, Dr J. Cuthbert, Dr R. J. Cuthbert, Dr B. R. Hillis, Dr G. Johnston, Dr J. W. Kerr, Dr A. W. Lees, Dr I. McHattie, Dr A. R. Russell, Dr B. H. R. Stack, Dr K. R. Urquhart, Dr E. R. Watson, Dr H. Yosef; Hammersmith: Dr C. G. McKenzie, Dr G. W. Poole, Dr P. Stradling; King's College: Dr D. M. Brinkley, Dr B. A. Hollis, Dr P. Hugh-Jones, Mr A. M. Macarthur; Middlesex, Ashford and Mount Vernon: Dr M. H. Bennett, Professor R. J. Berry, Dr W. C. D. Richards; Newcastle: Dr A. A. Brace, Dr R. A. L. Brewis, Dr W. K. Cowan, Dr R. G. B. Evans, Dr C. D. Jobling, Dr O. M. Koreich, Dr J. R. Lauckner, Dr P. O. Leggat, Dr I. MacLeod, Dr R. T. H. Shepherd, Dr B. J. Smith, Dr A. R. Somner, Dr E. A. Spriggs, Dr A. J. Watson; Norwich: Dr H. de C. Baker, Dr A. H. C. Couch, Dr B. D. W. Harrison, Dr A. W. Jackson, Dr W. F. Kerr, Dr M. J. Ostrowski, Dr J. H. Rack, Dr P. F. Roberts, Mr B. A. Ross; Oxford: Dr R. J. Adam, Dr J. M. Black, Dr W. S. Hamilton, Dr E. A. Hills, Dr E. O. S. Hope, Dr F. A. L. Kircher, Dr A. H. Laing, Dr D. J. Lane, Dr C. R. Newman, Dr A. O. Robson; Plymouth: Dr J. M. Brindle, Dr R. A. B. Drury, Dr A. C. Hunt, Dr W. Scarratt, Dr J. E. Scoble, Dr G. Sheers; SE RHA: Dr R. H. Andrews, Dr S. R. Drake, Dr M. Farquharson, Dr G. B. Forbes, Dr A. G. Gibson, Mr A. Golebiowski, Dr D. G. Jenkins, Dr J. Spencer Jones, Dr P. Matheson, Dr J. Pollert, Dr H. Wilson; Sheffield: Dr P. Huck, Dr M. Ross; Southampton: Dr P. E. Bodkin, Dr R. B. Buchanan, Dr R. C. Godfrey, Dr H. MacDonald, Dr G. M. Sterling, Dr A. E. Tattersfield, Professor D. H. Wright; Sunderland: Dr E. L. Feinmann, Dr K. A. Irvine, Dr S. Nariman, Dr J. H. Rolland Ramsay, Dr A. B. White; Teesside: Dr P. Ryan, Dr T. Skeoch, Dr H. I. Williams; Yorkshire: Mr L. Campbell-Robson, Dr N. Chakrabarti, Mr J. S. Davidson, Dr W. Davidson, Dr W. H. Helm, Professor C. A. Joslin, Dr H. S. Kellett, Dr A. J. King, Mr E. R. Lecutier, Dr D. Mackinnon, Dr D. K. Stevenson, Dr J. Stone, Dr G. W. Storey, Professor R. L. Turner, Dr A. J. Ward.

Dr K. F. W. Hinson was the reference pathologist for the study.

The trial was co-ordinated in the Medical Research Council Tuberculosis and Chest Diseases Unit by Dr L. E. Hill (by Dr D. J. Girling from October 1978) assisted by Mr P. M. Fayers and Mr R. J. Stephens.

### REFERENCES

BLEEHEN, N. M. (1979) Role of radiation therapy and other modalities in the treatment of small-cell carcinoma of the lung. In *Lung Cancer: Progress in Therapeutic Research*. Ed. Muggia & Rozenczwey. New York: Raven Press. p. 567.

BLEEHEN, N. M. (1980) The treatment of inoperable lung cancer by radiotherapy and chemotherapy. *Int. J. Radiat. Oncol. Biol. Phys.*, **6**, 1007.

COHEN, M. H., CREAVEN, P. J., FOSSIECK, B. E. & 5 others (1977) Intensive chemotherapy of small cell bronchogenic carcinoma. *Cancer Treat. Rep.*, **61**, 349.

DAWES, P. J. D. K. (1980) Results of a pilot study of wide field radiotherapy in the treatment of oat cell carcinoma of bronchus. *Clin. Radiol.*, **31**, 723.

FOX, W. & SCADDING, J. G. (1973) Medical Research Council comparative trial of surgery and radiotherapy for the primary treatment of small celled or oat celled carcinoma of the bronchus. 10-year follow-up. *Lancet*, ii, 63.

FOX, R. M., WOODS, R. L., BRODIE, G. N. & TATTERSALL, M. H. N. (1980) A randomised study: Small cell anaplastic lung cancer treated by combination chemotherapy and adjuvant radiotherapy. *Int. J. Radiat. Oncol. Biol. Phys.*, **6**, 1083.

HANSEN, H. H. (1980) Management of small-cell anaplastic carcinoma. In *Lung Cancer* 1980. Ed. Hansen & Rorth. Amsterdam: Excerpta Medica. p. 113.

HANSEN, H. H., SELAWRY, O. S., SIMON, R. & 4 others (1976) Combination chemotherapy of advanced lung cancer. A randomised trial. *Cancer*, **38**, 2201.

HANSEN, H. H., DOMBERNOWSKY, P., HANSEN, H. S. & RORTH, M. (1979) Chemotherapy *versus* chemotherapy plus radiotherapy in regional small-cell carcinoma of the lung. *Proc. American Association for Cancer Research and Amer. Soc. Clin. Oncol.*, **20**, 277.

HANSEN, H. H., DOMBERNOWSKY, P., HIRSCH, F. R., HANSEN, M. & RYGAARD, J. (1980) Prophylactic irradiation in bronchogenic small cell anaplastic carcinoma. *Cancer*, **46**, 279.

JOHNSON, R. E., BRERETON, H. D. & KENT, H. K. (1976) Small cell carcinoma of the lung: Attempt to remedy causes of past therapeutic failure. *Lancet*, ii, 289.

KREYBERG, L., LEIBOW, A. A. & UEHLINGER, E. A. (1967) Histological typing of lung tumours. *International Histological Classification of Tumours* No. 1. Geneva: W.H.O.

LEVISON, V. (1980) Pre-operative radiotherapy and surgery in the treatment of oat cell carcinoma of the bronchus. *Clin. Radiol.*, **31**, 345.

LIVINGSTON, R. B. (1979) Approaches to the control of central nervous system metastases in patients with small cell carcinoma of the lung. In *Lung Cancer: Progress in Therapeutic Research*. Ed. Muggia & Rozenczwey. New York: Raven Press. p. 587.

MEDICAL RESEARCH COUNCIL LUNG CANCER WORKING PARTY (1979) Radiotherapy alone or with chemotherapy in the treatment of small-cell carcinoma of the lung. *Br. J. Cancer*, **40**, 1.

OLDHAM, R. K. & GRECO, F. A. (1980) Small cell lung cancer: A curable disease. *Cancer Chemother. Pharmacol.*, **4**, 173.

PETO, R., PIKE, M. C., ARMITAGE, P. & 7 others (1977) Design and analysis of randomized clinical trials requiring prolonged observation of each patient. II. Analysis and examples. *Br. J. Cancer*, **35**, 1.

SALAZAR, O. M. & CREECH, R. H. (1980) The "State of Art" towards defining the role of radiation therapy in the management of small cell bronchogenic carcinoma. *Int. J. Radiat. Oncol. Biol. Phys.*, **6**, 1103.

SALAZAR, O. M., CREECH, R. H., RUBIN, P. & 5 others (1980) Half-body and local chest irradiation as consolation following response to standard induction chemotherapy for disseminated small cell lung cancer. *Int. J. Radiat. Oncol. Biol. Phys.*, **6**, 1093.


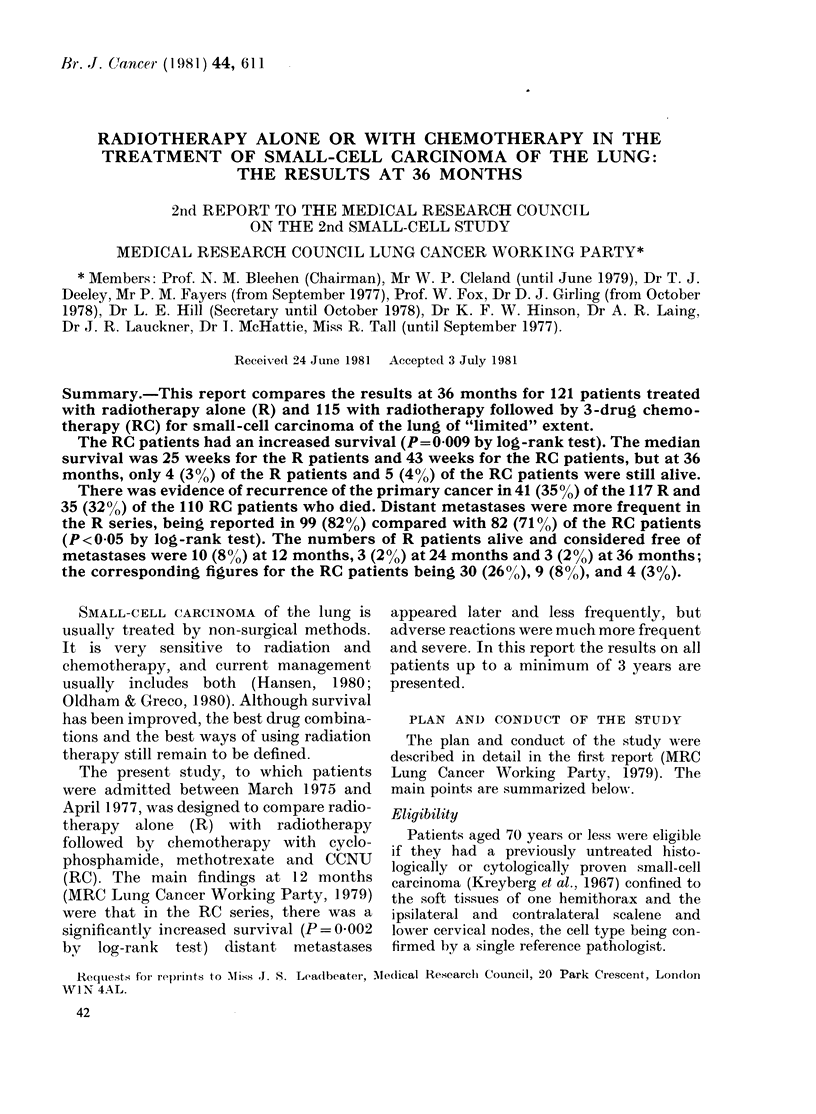

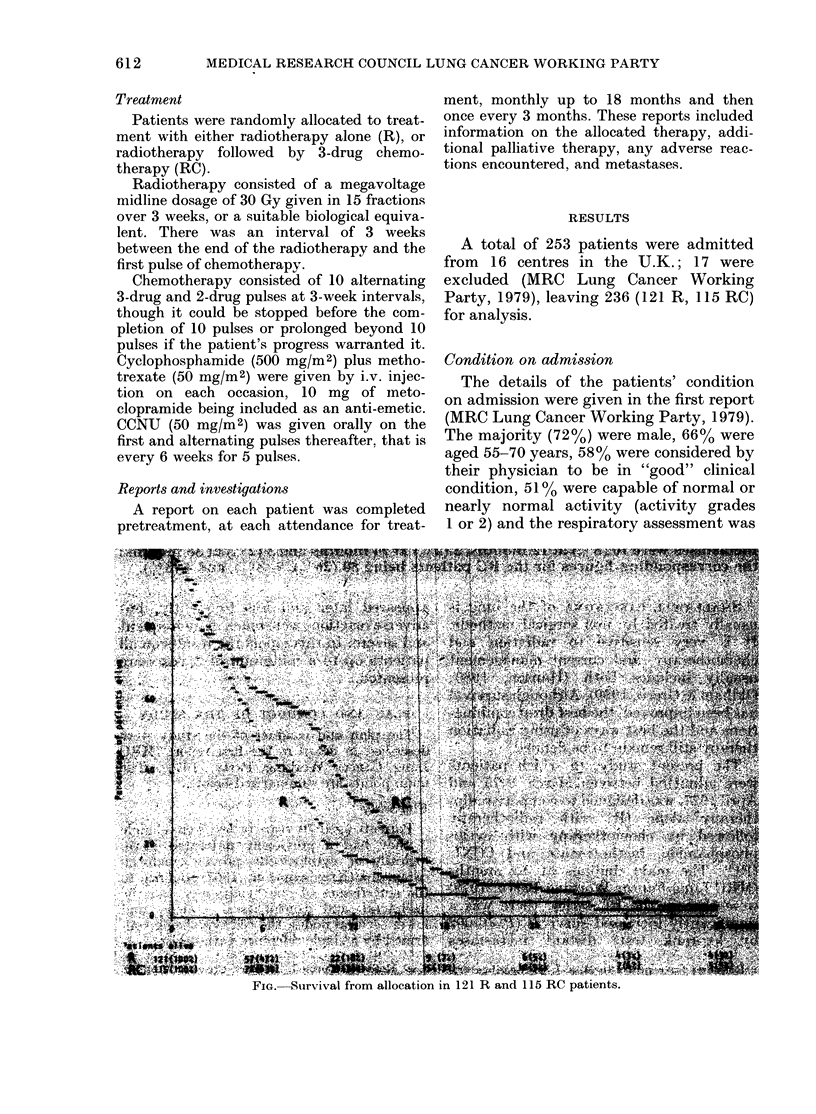

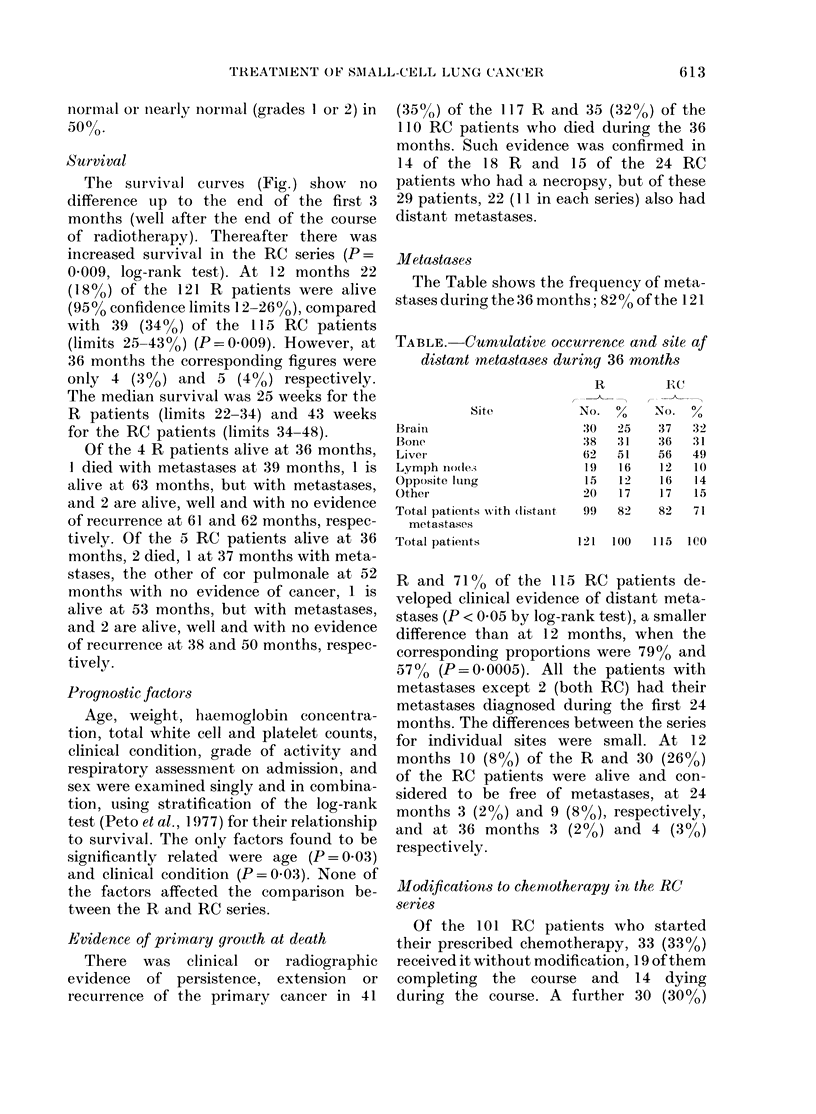

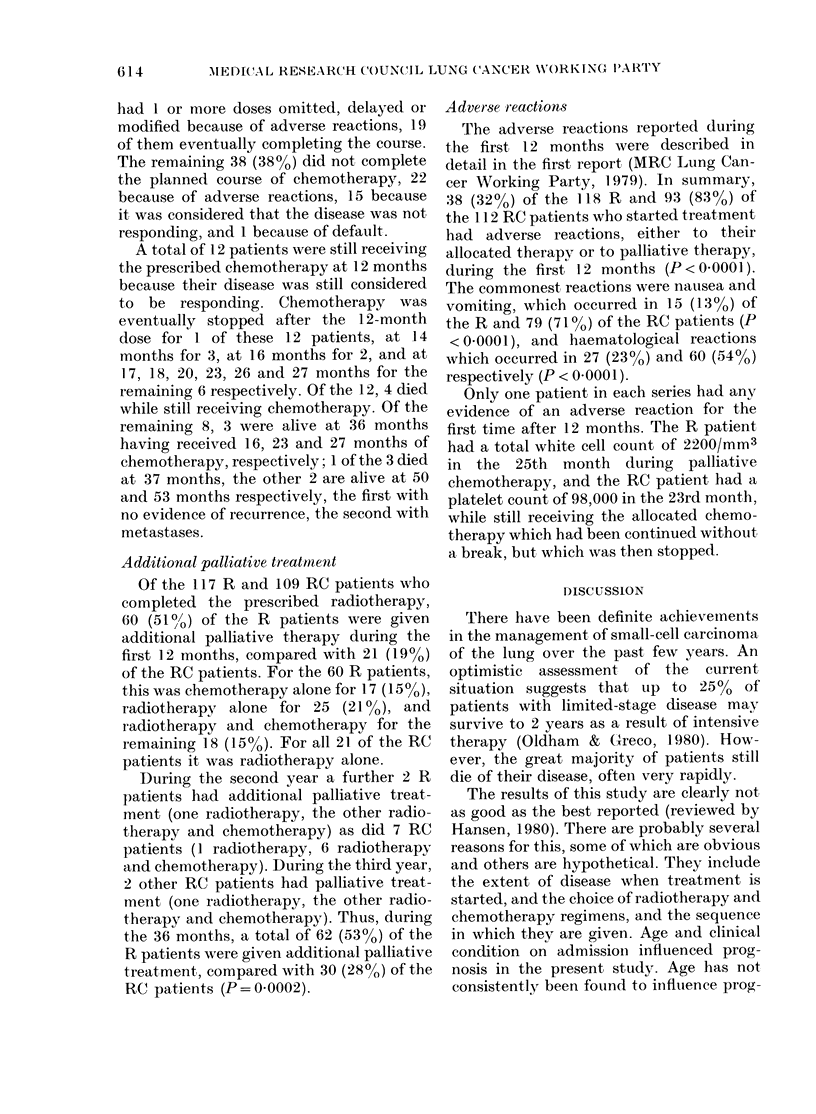

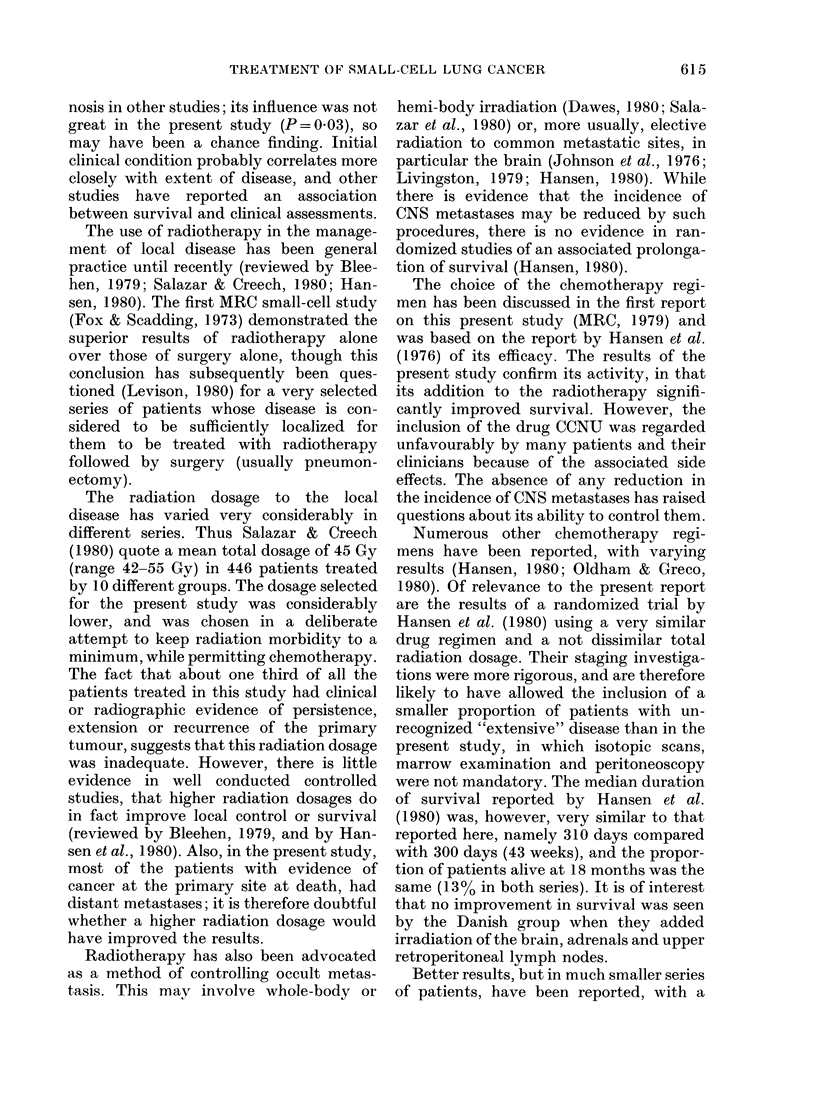

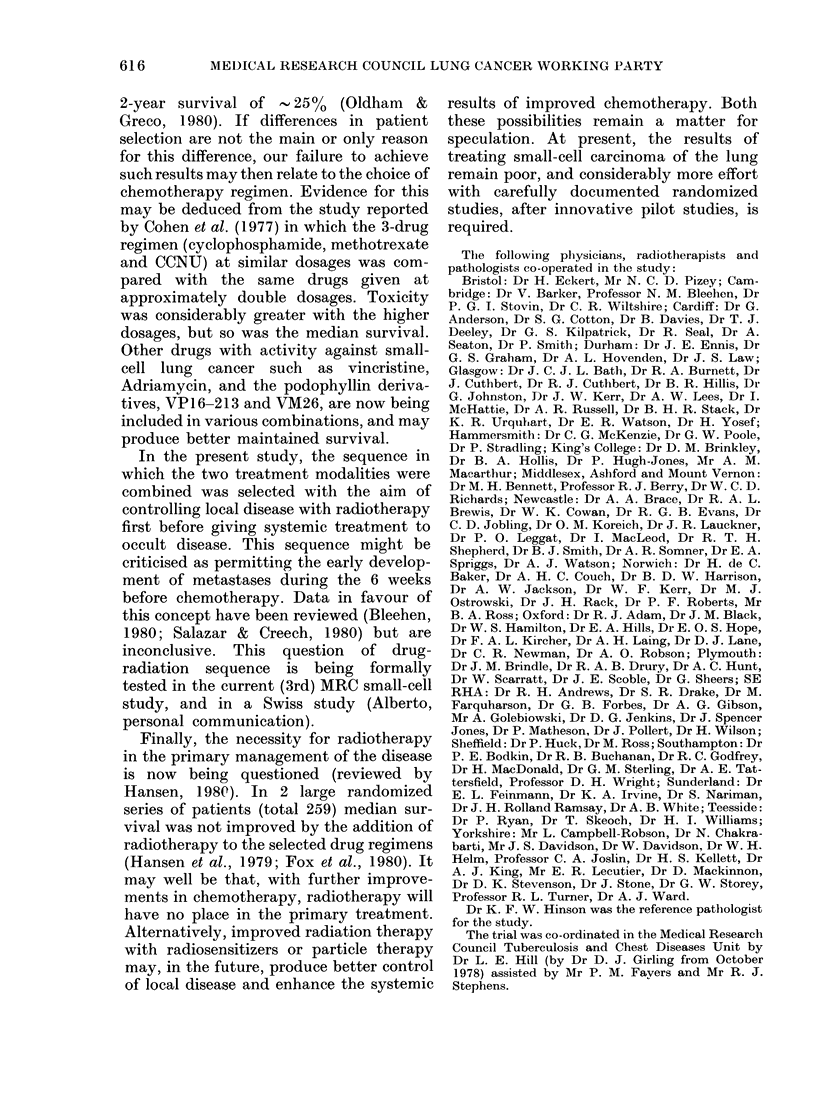

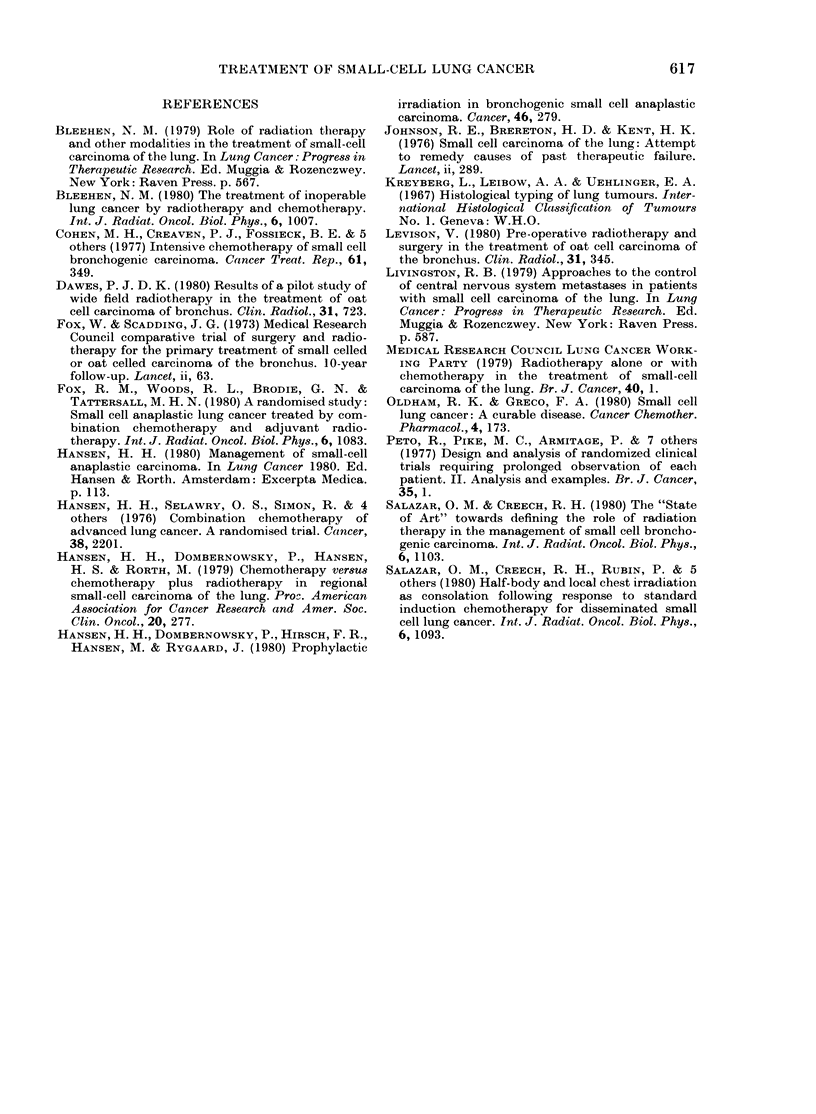


## References

[OCR_00722] Bleehen N. M. (1980). The treatment of inoperable lung cancer by radiotherapy and chemotherapy.. Int J Radiat Oncol Biol Phys.

[OCR_00727] Cohen M. H., Creaven P. J., Fossieck B. E., Broder L. E., Selawry O. S., Johnston A. V., Williams C. L., Minna J. D. (1977). Intensive chemotherapy of small cell bronchogenic carcinoma.. Cancer Treat Rep.

[OCR_00733] Dawes P. J. (1980). Results of a pilot study of wide field radiotherapy in the treatment of oat cell carcinoma of the bronchus.. Clin Radiol.

[OCR_00744] Fox R. M., Woods R. L., Brodie G. N., Tattersall M. H. (1980). A randomized study: small cell anaplastic lung cancer treated by combination chemotherapy and adjuvant radiotherapy.. Int J Radiat Oncol Biol Phys.

[OCR_00737] Fox W., Scadding J. G. (1973). Medical Research Council comparative trial of surgery and radiotherapy for primary treatment of small-celled or oat-celled carcinoma of bronchus. Ten-year follow-up.. Lancet.

[OCR_00770] Hansen H. H., Dombernowsky P., Hirsch F. R., Hansen M., Rygård J. (1980). Prophylactic irradiation in bronchogenic small cell anaplastic carcinoma. A comparative trial of localized versus extensive radiotherapy including prophylactic brain irradiation in patients receiving combination chemotherapy.. Cancer.

[OCR_00756] Hansen H. H., Selawry O. S., Simon R., Carr D. T., van Wyk C. E., Tucker R. D., Sealy R. (1976). Combination chemotherapy of advanced lung cancer: a randomized trial.. Cancer.

[OCR_00777] Johnson R. E., Brereton H. D., Kent C. H. (1976). Small-cell carcinoma of the lung: attempt to remedy causes of past therapeutic failure.. Lancet.

[OCR_00789] Levison V. (1980). Pre-operative radiotherapy and surgery in the treatment of oat cell carcinoma of the bronchus.. Clin Radiol.

[OCR_00808] Oldham R. K., Greco F. A. (1980). Small-cell lung cancer. A curable disease.. Cancer Chemother Pharmacol.

[OCR_00820] Salazar O. M., Creech R. H. (1980). "The state of the art" toward defining the role of radiation therapy in the management of small cell bronchogenic carcinoma.. Int J Radiat Oncol Biol Phys.

[OCR_00827] Salazar O. M., Creech R. H., Rubin P., Bennett J. M., Mason B. A., Young J. J., Scarantino C. W., Catalano R. B. (1980). Half-body and local chest irradiation as consolidation following response to standard induction chemotherapy for disseminated small cell lung cancer: an Eastern Cooperative Oncology Group pilot report.. Int J Radiat Oncol Biol Phys.

